# Development of an Intelligent System for the Monitoring and Diagnosis of the Well-Being

**DOI:** 10.3390/s22249719

**Published:** 2022-12-12

**Authors:** Lizeth-Guadalupe Machado-Jaimes, Martin Rogelio Bustamante-Bello, Amadeo-José Argüelles-Cruz, Mariel Alfaro-Ponce

**Affiliations:** 1 Tecnologico de Monterrey, School of Engineering and Science, Monterrey 64849, Mexico; 2 Instituto Politécnico Nacional, Centro de Investigación en Computación, Mexico City 07738, Mexico; 3 Tecnologico de Monterrey, Institute of Advanced Materials for Sustainable Manufacturing, Monterrey 64849, Mexico

**Keywords:** smart monitoring systems, health monitoring, internet of things

## Abstract

Today, society is more aware of their well-being and health, making wearable devices a new and affordable way to track them continuously. Smartwatches allow access to daily vital physiological measurements, which help people to be aware of their health status. Even though these technologies allow the following of different health conditions, their application in health is still limited to the following physical parameters to allow physicians treatment and diagnosis. This paper presents *LM Research*, a smart monitoring system mainly composed of a web page, REST APIs, machine learning algorithms, psychological questionnaire, and smartwatches. The system introduces the continuous monitoring of the users’ physical and mental indicators to prevent a wellness crisis; the mental indicators and the system’s continuous feedback to the user could be, in the future, a tool for medical specialists treating well-being. For this purpose, it collects psychological parameters on smartwatches and mental health data using a psychological questionnaire to develop a supervised machine learning wellness model that predicts the wellness of smartwatch users. The full construction of the database and the technology employed for its development is presented. Moreover, six machine learning algorithms (Decision Tree, Random Forest, Naive Bayes, Neural Networks, Support Vector Machine, and K-nearest neighbor) were applied to the database to test which classifies better the information obtained by the proposed system. In order to integrate this algorithm into *LM Research*, Random Forest being the one with the higher accuracy of 88%.

## 1. Introduction

In our bodies, everything is connected, and emotions can alter the welfare of a person. According to the World Health Organization, health is not only the lack of disease but the combination of physical, mental, and social well-being [1]. Most of the population focuses on maintaining physical health by forgetting that mental health can have an intense relationship with physical health [2]. Among the instruments for evaluating physiological conditions, we can observe various evaluation modalities, including self-register, clinical classification scales, indirect observation methods, and evaluations of significant samples. For the mental condition, some attempts have been made to create tests covering anxiety and depression and their relationship with quality of life and health. In this sense, (Quality of Life Enjoyment and Satisfaction) Q-LES-Q and the Depression Anxiety Stress Scale 21 (DASS-21) questionnaires allow researchers to easily obtain measurements of the degree of enjoyment and satisfaction experienced by subjects in various areas of daily activity [3,4].

Mental health can have an intense relationship with physical health. Having the disorders stated previously can change the way you live. Anxiety and sadness/depression reactions that are too severe or persistent over time can cause behavioral changes, such as forgetting healthy habits and the development of addictive or unhealthy behaviors that endanger our health. These emotional reactions maintain intense levels of physiological arousal, and these high levels may be associated with a certain degree of immunosuppression, which makes people more vulnerable to the development of infectious or immunological diseases [5]. More particularly, as a negative emotion, anxiety can trigger three main responses in our body: physiological, cognitive, and motor. Thus, two physiological systems are involved: the autonomic nervous system and the neuroendocrine system. This system activates in response to a situation and is responsible for heart rate elevation, increased muscle tension, and blood pressure, among other effects [5].

In addition, the strong relationship between physical and mental health has demonstrated that physical exercise induces structural and functional changes in the brain [6]. Consequently, several studies support that exercise routines are associated with fewer symptoms of depression, lower anxiety, and greater emotional well-being. Even though the positive effects of exercise on the mental state are well-known, the mechanism that triggers those benefits remains unclear [7]. In order to study these benefits, several research groups have employed wearables to measure physical exercises to asses different benefits to the user’s physical health through machine learning (ML) and statistical algorithms for analyzing the massive data these devices can provide [8].

Ghayvat et al. developed a lifestyle monitoring for assisted living that aims to diagnose conduct variation in the frequent behavior modeling for an individual in ambient assisted living; for it, they propose a novel activity learning model that employed observations that were tagged as wellness indices [9]. Marques et al. [10] introduce iAmb, a system for indoor ambient quality monitoring through IoT that users can access via the web, smartphone and smartwatch. Chuah [11] presents a study about the influence of smartwatches on people’s behaviour and changes in lifestyle. Casaccia et al. [12] introduces an assessment for sensing devices that allow for continuous remote monitoring to improve people’s well-being; the system considers perceptions, needs, and preferences.

This project presents an intelligent system that monitors people’s physical and mental parameters to prevent a possible well-being crisis. In order to do this, monitoring will be done through a combination of psychological and mental health data compilation that will be processed via ML algorithms for a prediagnosis of user well-being for the system development; wearable, IoT and cloud computing were employed. The document is organized as follows: Section 2 introduces the different applications of wearable devices in health. Section 3 gives a full description of the Garmin smartwatches’ database, how it was tagged through psychological questionnaires, and how information is processed and classified by MLalgorithms. Section 4 presents the results achieved by the different stages of this work. Finally, Section 6 gives the conclusions of this project.

## 2. Wearable Health Monitoring Systems

Zheng [13] states that wearables are changing traditional diagnostic methods by making new medical devices with remote wearable and portable functions. Patients now have the opportunity to reduce the time spent in the hospital thanks to health monitoring systems. By introducing these wearable healthcare systems, it is possible to improve health status and contribute greatly to the development of medical technology by collecting human health information [14].

In addition, according to Zheng, all wearable health devices consist of the following elements:

1. Integrated Circuits
2. Display
3. Signal Processing
4. Wireless Communication: (a) NFC, (b) Wi-Fi and (c) Bluetooth
5. Receivers: (a) Smartphones and (b) Laptops
6. Power sources: (a) Solar energy and (b) Nanogenerators

Several off-the-shelf wearables have the following sensors: body motions, reparations rate, and blood pressure. These are important because they can provide early diagnosis of diseases.

### Health Tracking Research at the Edge

Various projects merge health monitoring with the internet of things (IoT) [15]. Stephanie B. Baker in [16], states that IoT can improve health systems in many branches of the medical field because IoT healthcare systems have been developed for specific purposes [17], including rehabilitation [18], diabetes management [19], assisted ambient living (AAL) for the elderly persons [20], and more.

In a health care system, the instruments that sense the vital signals will be connected to a computer that will process all the data the sensors send [21]. As Stephanie B. stated, diabetes is one of the diseases that smart health systems should focus on monitoring because, until now, it has been incurable. Therefore, using the IoT, Shih-Hao Chang developed an interactive health system with real-time communication between patients and doctors. The project consists of three devices: a General Packet Radio Service (GPRS) blood-glucose monitor (BGM), a telecare Android and iOS application for caregivers, and a cloud server platform to process all the data given by the sensors mentioned above [22].

Moreover, there has been more research into making these healthcare systems more affordable. Kartikke Uplenchwar developed a monitoring system using the IoT, and a Raspberry Pi and an Arduino were employed for the information processing. This system will measure electrocardiogram (ECG), Pulse rate, Weight, Temperature and Position detection using wearable sensors. The sensors are conceded to the main node or server (Raspberry Pi and Arduino) that processes the information via the internet [23].

Similarly, Kirankumar implemented a low-cost Web-Based Human Health Monitoring System using only Raspberry Pi 2. The Raspberry Pi is connected to the internet, so it can be processed and displayed on a web page [24]. The principal parameters it senses are blood pressure rate, temperature, heartbeat rate, alcohol sensor, ECG sensor, sound sensor, and EMG sensor to determine the patient’s stress level and the video camera to collect the patient’s live streaming video.

With the description of the applications mentioned above, wearable devices enable the collection of enormous amounts of physiological data, which can be utilized to better understand a person’s health and behaviour [25,26]. However, there are no open datasets within the literature that offer psycho-physiological data. In order to target the lack of information, Alessio Rossi presented The Multilevel Monitoring of Activity and Sleep in Healthy people (MMASH) dataset, which provides 24 hr of continuous psycho-physiological data [27]. This dataset tracked beat-to-beat data, triaxial accelerometer, anxiety status, stress events, emotions, and sleep quality of 22 healthy subjects. Even though databases with multiple parameters associated with well-being have been created, there are still issues with how data are processed for expert analysis. Furthermore, the amount of data is huge, making it hard to process for the medical specialist, making intelligent systems a possible solution aiding specialists in diagnosing and treating patients.

## 3. Materials and Methods

**Figure 1:**
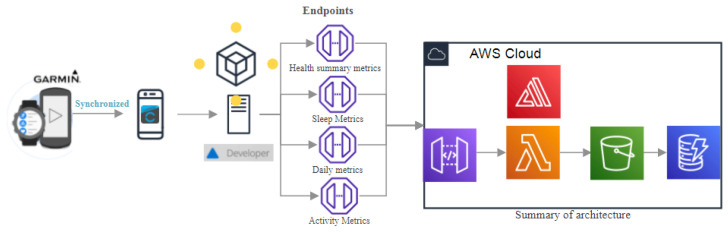
Health tracking system.

This work interweaves physical and psychological parameters obtained by a smartwatch to determine a person’s overall well-being. For it, a communication API integrated into Garmin’s developer portal and smartwatches make up the materials used in the research. Users can use the website, which is connected to the API, can create a connection endpoint, a database and a dashboard to display and request personal information. In addition, all data collected by the API are stored in a cloud database for further processing, employing ML in Amazon Web Services and generating a wellness report. The required technologies or services for the project creation are shown in Figure 1.

### 3.1. Relationship between Mental Health and Physical Well-Being through Smartwatch

An analysis was performed to know how stress, anxiety, and depression influence the main vital signals of the body. Anxiety and stress are natural reactions of the body, but when threats or uncertainty become too intense or too frequent, they can cause mental health disorders with symptoms such as shortness of breath, insomnia, nausea, sweating, loss of appetite, dizziness, fatigue, upset stomach, heart pounding, and inability to meet people or leave the house. For these reasons, the primary measurements of a smartwatch, particularly from the Garmin brand, are: wrist-based heart rate (constant, every second), daily resting heart rate, all-day stress, sleep (light sleep, deep sleep, REM sleep) [28], calories burned, distance traveled, step counter, intensity minutes, stress. Furthermore, a person’s physical characteristics may need to be considered in the correlation between mental and physical health; these characteristics may be weight, height, and age.

### 3.2. Garmin Developer Portal as a Tool for Database Construction

The Garmin Developer Portal was utilized to gather the physical parameters required for the project. The Garmin Connect API grants authorized third-party developers access to this extensive data set in exchange for the user’s consent. In addition to the traditional API access, Garmin also provides a service for synchronizing its servers with the developer, putting the original data archive (JSON) at its disposal via the PUSH method. Cloud-to-cloud interactions are possible with the Garmin Connect APIs. Real-time data and a direct connection between a developer’s web app and Garmin wearables are available by using Garmin Health SDKs. For the development of the database employed for this work, the data obtained by the smartwatches were tagged through well-known psychological questionnaires, allowing to determine the user’s well-being.

The DASS 21, the Ryff Psychological Well-Being Scale and the Q-LES-Q were the questionnaires employed for this work. The DASS-21 is an instrument to screen for symptoms at different levels of depression, anxiety, and stress [29]. Ryff Psychological Well-Being Scale associates psychological well-being with a psychological state by establishing an individual’s psychic activity with his way of adapting to the intrinsic and extrinsic needs of the physical and social environment. Q-LES-Q is a self-reporting measure tool assessing mental health, taking into account the person’s daily life [30]. In order to train the intelligent algorithm presented in the next sections, every third day, each user completed one of these questionnaires, and by doing so, they contributed to creating a new entry in the database. These responses will be linked to the physical metrics collected from the smartwatch on that particular day.

### 3.3. Elaboration of Project’s API (*LM Research*)

The creation of a cloud-based web health API (website) named *LM Research* served as the foundation for this entire project, as it is here that both the physical data provided by the watch and the psychological data of each user are received. Similarly, on the web API of the project, the received data are cleaned and preprocessed to create a filtered No SQL database, which is being analyzed using various ML algorithms. *LM Research* is composed of three major components: the webpage, the API gateways used for Garmin connection, and the web service used to process all the information received from Garmin and the webpage.

AWS’s API gateway service generates each endpoint’s URL. API Gateway is in charge of accepting and processing requests between two or more APIs. Each endpoint generated in AWS is specified in the endpoint section of the Garmin developer platform. Moreover, each endpoint has a lambda function linked to it to save the received JSON in the S3 bucket.

### 3.4. Amazon Web Services

AWS was used to develop the API described in this project. Various AWS services were used to develop *LM Research*. These services were used in conjunction with the API components. Amplify, in particular, was the AWS service encapsulating the entire project because it is where the connections of the other AWS services were established. Furthermore, all data received via the API gateway were stored in Amazon Simple Storage Service (S3) and processed in Amazon DynamoDB. As a result, the development of a smart system that continuously assists users in monitoring their well-being is an everyday part of life. Each service and technology will be thoroughly explained in the following components of *LM Research*.

**Figure 2:**
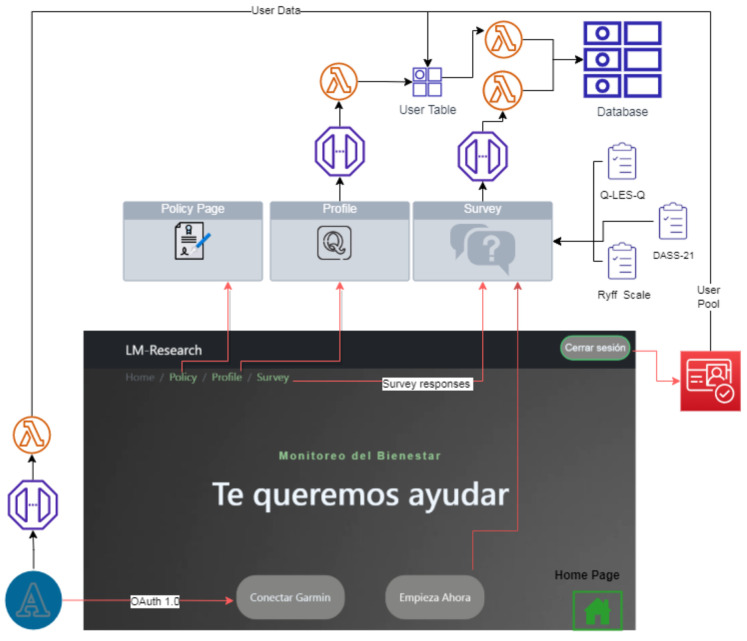
Pages and services of *LM Research*.

**Figure 3:**
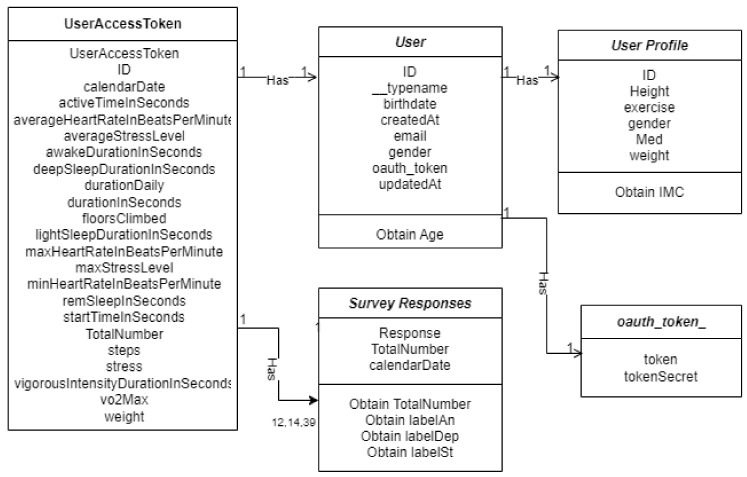
Front-End and Back-End of *LM Research*.

**Figure 4:**
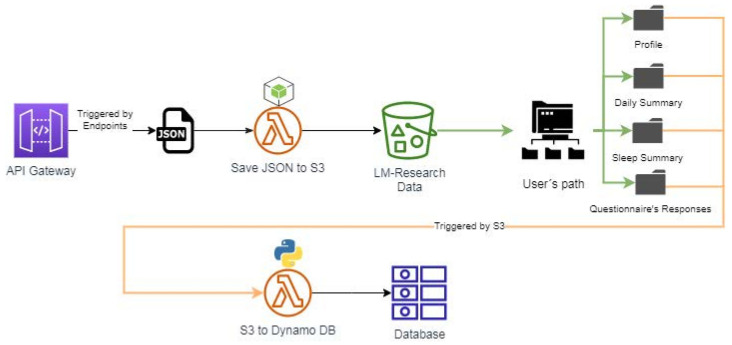
Lambda Functions for reception of data.

**Figure 5:**
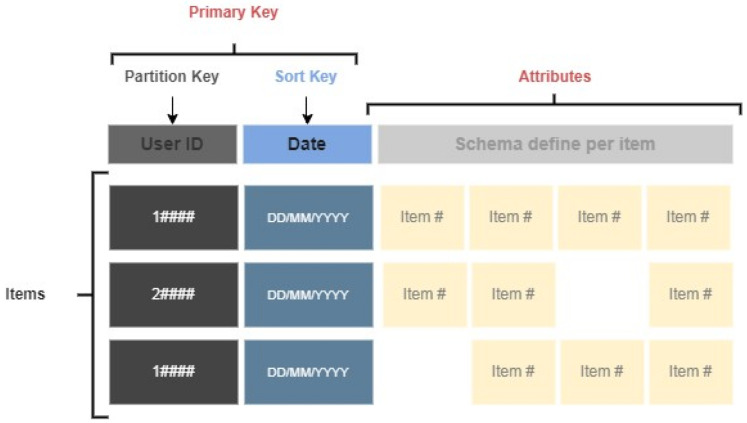
Representation of database.

- Web Page: A dynamic web page is a computer application that uses databases to load information. The content of these pages may vary depending on the interaction of the web visitor. A total of four pages were programmed for the elaboration of the website. The four pages were programmed in the React application. The following Figure 2 explains which services are used on each page.
  For the user login and account, registration pages were designed to connect with Amazon Cognito via Amplify. The integration of Amazon Cognito and the code of the *LM Research* web page provided AWS Back-End functionality for authentication and authorization workflows. Congnito used a lambda function to create a table in DynamoDB that contained all the data for each user. In addition, the user’s table was used in other Lambda functions to create the main database. Figure 3 demonstrates the connections between the front end and the back end with the project’s API and the technologies employed at each level.
  The responses are saved by integrating different services: the API Gateway, which posts the responses via RESTful method in a JSON format, and a lambda function, triggered when the specified endpoint receives the JSON. The lambda function saves the responses in the database’s Main table in DynamoDB. Furthermore, each response to the users’ questionnaires on the website is saved in the same way: another lambda function is triggered by an endpoint in the API gateway. The responses are saved in the main DynamoDB table by this function. The questions needed are:
  1. Do you take medications for stress, anxiety, or depression?
  2. Do you exercise?
  3. How much do you weigh?
  4. What is your height?
  The answers to these questions provided us with insight into the user’s way of life and its physical complexion.
- Acquisition of physical parameters from Garmin wearable. The main distinction is that a Web service enables two devices to interact via a network, whereas an API is an interface. For the connection of the project’s API (*LM Research*) to Garmin, it was necessary to set up an account on Garmin’s portal and post the basic information about *LM Research*. A cloud-based Web API in AWS was implemented for accessing users’ cloud-based fitness data through the Garmin Developer Portal (GDP). The platform can be found at the following link: https://developerportal.garmin.com, accessed on 15 July 2021.
- Data Processing. All information that receives *LM Research* must be routed through the API gateway service, which triggers several Lambda functions that save the received JSONs in S3 buckets or directly to the main table in DynamoDB with all users’ data sorted by day and user’s tokens. The raw data received at the multiple endpoints contain many unnecessary data and parameters that are not relevant to the project. Henceforth, cleaning up and addressing the prior issue is required so that the ML algorithm can accurately “understand” the data. The lambda functions are responsible for cleaning and filtering the raw data. Specifically, three Lambda functions are used and are shown in Figure 4.
- Database. The database was developed in DynamoDB. The table’s primary attributes, partition, and sort keys. The table has a primary key, which cannot be changed once it is set. This key identifies each item in the table uniquely. Therefore, no two entries can have the same key. This table’s key comprises two types of keys: partition(userId) which refers to the user’s ID, and sort key (date), which is the date the data were received. When querying the data, the composite primary key provides more search options. The Figure 5 table indicates how the table was built, with the attributes comprised of variables retrieved from the smartwatch, such as heart rate, sleep, steps, and so on, and the mental parameters obtained for the questionnaires.

### 3.5. Machine Learning as a Tool for the Well-Being Diagnosis

Once the database was created, a ML algorithm was proposed to automatically diagnose the user’s well-being; this stage and the process of the data are described next.

#### 3.5.1. Feature Selection

Dimensionality reduction is linked to feature selection, which is critical in machine learning, whether for pattern recognition or classification, to optimize the overall process. The process of identifying the most essential or relevant data collection features to increase the predictors’ classification performance is known as feature selection. For the classification performance, various techniques were employed to extract the data, including:

1. F-mutual: It determines the mutual information between two random variables to assess their interdependence.
2. F-classif: Compute the ANOVA F-value.
3. chi-squared: It calculates the chi-squared statistics for each non-negative feature

#### 3.5.2. Machine Learning for Well-Being Classification

To classify the user’s well-being, the following six algorithms were applied:

1. Decision Tree: Given a set of data, logical construction schemes are generated (rules).
2. Random Forest: is a collection of classifiers comprised of numerous decision trees. Trees are trained on subsets of the original dataset, and the average is utilized to increase accuracy and control over-fitting.
3. Naive Bayes: It is a probabilistic classifier based on Bayes’ theorem, with all qualities assumed to be independent.
4. Neural networks: It employs a series of neuronal layers to solve problems that cannot be solved linearly (the primary challenge of basic perception).
5. Support Vector Machine (SVM): This method is based on the notion of a hyperplane. When there are n observations, each with p predictors and a response variable with two levels, hyperplanes can be used to design a classifier that predicts to which group an observation belongs based on its predictors.
6. K-nearest neighbors (KNN): It classifies depending on the information provided by the prototype set, determining their proximity to them based on their attributes. It calculates the distance between each K neighbors to create the classification.

**Figure 6:**
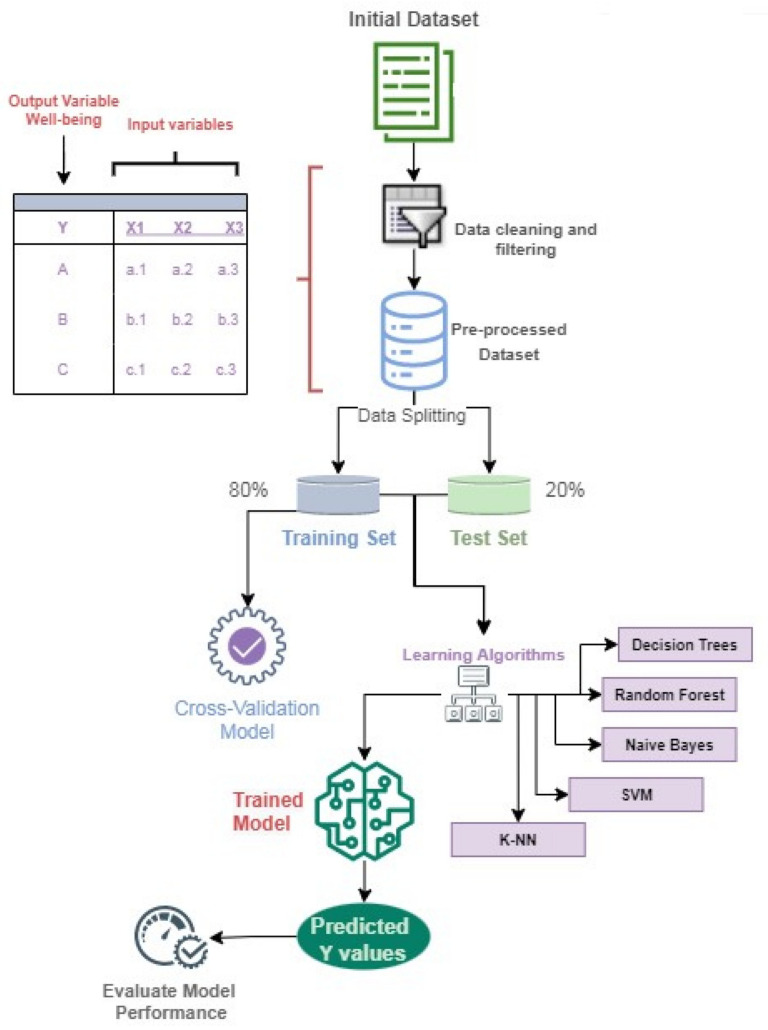
Process used for classification algorithms.

The ML process is displayed in Figure 6. After obtaining the final dataset, data pre-processing was made: A filter will be designed to evaluate that all the entries were complete and that there were no missing data in any of the 20 variables, so if the user did not synchronize the clock with the phone one day, data for that day would be missing. Next, feature extraction will be applied to optimize the intelligent algorithm. Several approaches will be used to identify the variables that most influence the model, and k-fold cross-validation, with k = 5, will be implemented to validate the four ML algorithms. Finally, decision tree, random forest, Naive Bayes, SVM, and KNN algorithms were implemented and tested using confusion matrices to determine the accuracy of each and choose the best one.

## 4. Results

### 4.1. Web Page

*LM Research* is a dynamic web page generated on demand; the content will change depending on the user, mostly because it graphs their personal physical and mental parameters, which have been measured since they registered on the web page. The web page can be consulted at the following link: https://master.d2geuvhg2er1e6.amplifyapp.com/welcome.

An HTML, CSS, and JavaScript code coder was employed to develop *LM Research*. The React library was specifically used. React is a free and open-source JavaScript library for building user interfaces.

The following pages were programmed:

1. Home Page: It serves as the website’s cover and explains the project’s goal.
2. Profile Page: On this page, the user must answer a series of one-time questions regarding their lifestyle.
3. Policy Page: This page displays the user agreement, which details how the project will utilize its data.
4. Connect with Garmin: This is where the OAuth connection between Garmin and the API is established. This link will take you to the Garmin OAuth page.
5. Survey page: On this page, users will complete surveys. The surveys will be displayed at random among the three categories of questionnaires.
6. Report: Users will see their daily physical parameters and well-being plotted on this page from the first day they registered for the study. The following graphs will be displayed: (a) well-being classification, (b) average heart rate in beats per minute, (c) average stress level, (d) deep sleep duration in seconds, (e) light sleep duration in seconds, (f) maximum stress level, (g) maximum heart rate in beats per minute, (h) steps.

### 4.2. Database

**Figure 7:**
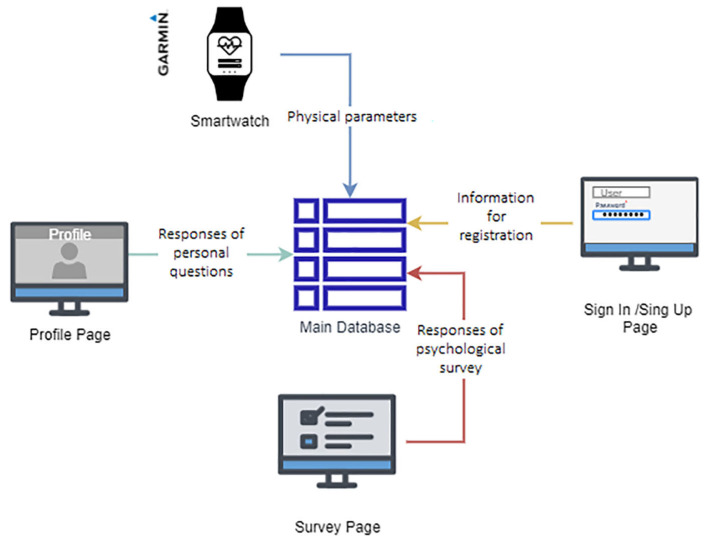
Composition of the database.

Four separate sources as shown in Figure 7 were required to generate the database: the physical parameters recorded on the profile page, the sociocultural data recorded on the profile page, the information provided by each user at the time of registration, and the responses to the three distinct questions. The last three were gathered from the different pages on the website.

Each day, new information in JSON format is received from these sources, which is cleaned and filtered before being saved in the dynamo table, the database’s primary table.

The database comprises 36 columns, of which 35 represent the extracted features, and the remaining is used to label each database entry.

1. userAccessToken
2. calendarDate
3. activeTimeInSeconds
4. age
5. averageHeartRateInBeatsPerMinute
6. averageStressLevel
7. awakeDurationInSeconds
8. deepSleepDurationInSeconds
9. depression
10. durationDaily
11. durationInSeconds
12. exercise
13. floorsClimbed
14. gender
15. height
16. id
17. idQuest
18. IMC
19. labelAn
20. labelDep
21. labelSt
22. lightSleepDurationInSeconds
23. maxHeartRateInBeatsPerMinute
24. maxStressLevel
25. med
26. minHeartRateInBeatsPerMinute
27. remSleepInSeconds
28. startTimeInSeconds
29. steps
30. stress
31. TotalNumber
32. vigorousIntensity
33. vo2Max
34. weight

The labeling is created with the database’s Total Number feature, representing the user’s score on a certain date’s questionnaire. As a result, the training database now contains 35 characteristics shown in the previous list (Section 4.3) and 96 data items from 22 people. To be more precise, each entry records the 35 attributes acquired from the four sources indicated before (Section 4.2) from the 22 users each day.

### 4.3. Pre-Processing

The majority of the parameters are collected from seven different models of Garmin smartwatches among the 22 users. Because of the variety of models, the parameters measured by each model can vary. As a result, the first data filter needed to be established, which consisted of extracting the data that were measured “*vo2Max*” and “*floorsClimbed*” parameters all the wearables shared. Furthermore, not all watches measure “*vigorousIntensity*”. The variable “*durationDaily*” is merely a variable that specifies the seconds the watch has recorded all the body parameters; it is not connected with well-being, so it will not be taken into account when constructing the final database. Moreover, the two separate identifiers and the calendar date that the table held were no longer needed to train the database, and “*ID*”, “*calendarDate*” and “*userAcessToken*” were removed. The variables “*label* stress”, “*label anxiety*” and “*label* depression” (variables derived from type 0 questionnaire responses) were removed. As a result, the following attributes were removed from the database:

- vo2Max
- floorsClimbed
- vigorousIntensity
- durationDaily
- CalendarDate
- ID
- userAcessToken
- labelAn
- labelDep
- labelSt

Because there are 21 different variables, a reduction of variables is necessary to optimize the training phase of the ML algorithm.

1. activeTimeInSeconds
2. age
3. averageHeartRateInBeatsPerMinute
4. averageStressLevel
5. awakeDurationInSeconds
6. deepSleepDurationInSeconds
7. durationInSeconds
8. exercise
9. gender
10. height
11. IMC
12. lightSleepDurationInSeconds
13. maxHeartRateInBeatsPerMinute
14. maxStressLevel
15. med
16. minHeartRateInBeatsPerMinute
17. remSleepInSeconds
18. startTimeInSeconds
19. steps
20. TotalNumber
21. weight

### 4.4. Encoding the Response Variable

The crucial aspect of this study is the response variable, which is unwellness or lack of well-being. The answers to the three different surveys, the *TotalNumber* variable, were divided into three classes to classify the person’s well-being. Because *TotalNumber* has values ranging from 0 to 174, a transformation will be required to generate the well-being variable. If the *TotalNumber* falls within a certain range, the wellness will be 0, 1, or 2. As a result, the algorithm’s output will classify the person’s wellness into three groups, and the output variable will be transformed into ordinal data.

Different operations (7) were performed to categorize the range in which is category0, category1, and category2 depending on the score ( *TotalNumber*) of all the questionnaires in the dataset. First, it was necessary to establish the section in which the resultant score will be divided depending on the answers and the type of questionnaire.

(1) $$\begin{array}{c} Sec=\frac{max\left(TotalNumber_{X}\right)-min\left(TotalNumber_{X}\right)}{3}\; \end{array}$$

where *TotalNumberX* is the score of the responded survey and which *X* represents the following questionnaires:

1. DASS-21 (type 0)
  (a) Number of questions: 21
  (b) Minimum score: 0
  (c) Maximum score: 63
2. Quality of Life Enjoyment and Satisfaction Questionnaire (Q-LES-Q)
  (a) Number of questions: 14
  (b) Minimum score: 14
  (c) Maximum score: 70
3. Ryff Psychological Well-Being Scale
  (a) Number of questions: 29
  (b) Minimum score: 29
  (c) Maximum score: 174

Then depending on the type of survey, the ranges will be created to categorize the well-being of the users in the following ways.

**Questionnaire type 0**

(2) $$\begin{array}{c} Range_{0}=[min\left(TotalNumber_{0}\right),\\ \;\;min(TotalNumber_{0})+sec]\; \end{array}$$

(3) $$\begin{array}{c} Range_{1}=[min\left(TotalNumber_{0}\right)+sec,\\ \;\;\;min(TotalNumber_{0})+2*sec]\; \end{array}$$

(4) $$\begin{array}{c} Range_{2}=[min\left(TotalNumber_{0}\right)+2*sec,\\ \;\;\;\;\;\;\;max(TotalNumber_{0})]\; \end{array}$$

**Questionnaire type 1,2**

(5) $$\begin{array}{c} Range_{2}=[min\left(TotalNumber_{0}\right),\\ \;\;min(TotalNumber_{0})+sec]\; \end{array}$$

(6) $$\begin{array}{c} Range_{1}=[min\left(TotalNumber_{0}\right)+sec,\\ \;\;\;min(TotalNumber_{0})+2*sec]\; \end{array}$$

(7) $$\begin{array}{c} Range_{0}=[min\left(TotalNumber_{0}\right)+2*sec,\\ \;\;\;\;\;\;\;max(TotalNumber_{0})]\; \end{array}$$

**Figure 8:**
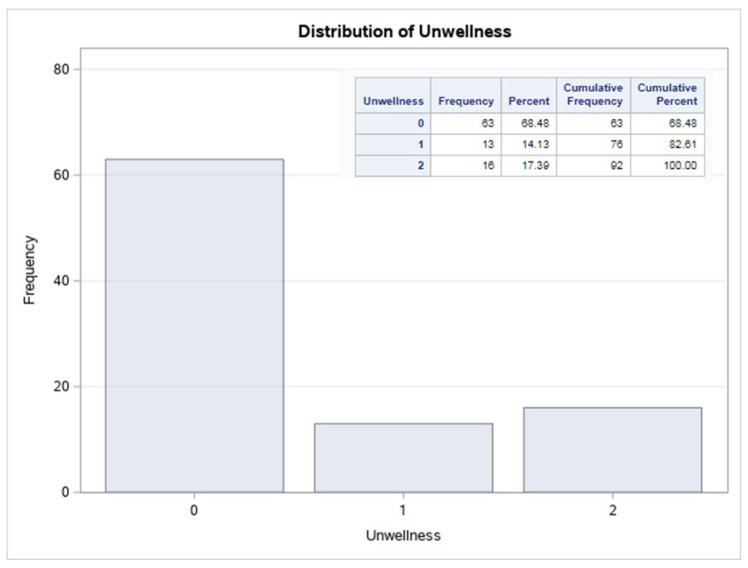
Distribution of well-being.

After data coding, three categories of well-being were defined. Because the variable *TotalNumber* was converted from a numerical to a categorical variable, the name was changed to **Unwellness** to better indicate the categories (0,1,2) that these parameters have. Figure 8 represents how those categories are distributed in the final database.

As illustrated in the graph, the response variable is unbalanced between people with high to low well-being, being 0 in the variable **Unwellness** high well-being. Most classifier learning algorithms that presume a typically balanced distribution have trouble with a data set that has an unbalanced class distribution. As a result, the training of the algorithm became more difficult, and feature selection was required to optimize it.

#### 4.4.1. Data Analysis

For the analysis, it is important to observe each variable used in the final data set. The variables were divided into two types; the response variable, which is unwellness or lack of well-being and has three values; 0 for excellent well-being, 1 for average well-being, and 2 for poor well-being. The second type is the explanatory variables or independent variables, which are 21 different variables mentioned in the preceding Section 4.3. Only gender and activity are categorical variables, having values of 0 and 1, while the remaining variables are numerical.

**Figure 9:**
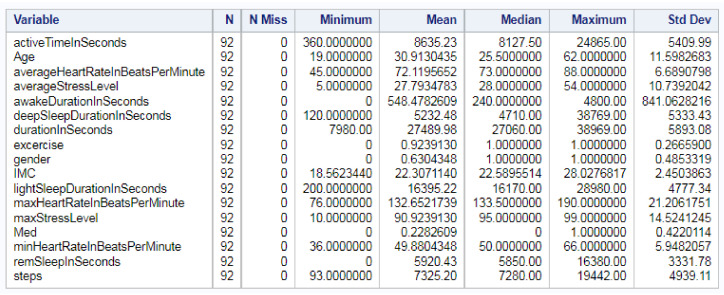
Descriptive Statistics for Numeric Variables.

Measurements that summarize the information from all variables used to train the data set are displayed in Figure 9. These measurements will be used later to standardize all ML algorithms’ inputs.

#### 4.4.2. Relationship of Variables

Some variables are related to each other; for example, well-being could be related to age: how active the person is, quality of periods of sleep, etc. All these factors can influence the result of daily well-being, as is stated by the research made by [31]. Moreover, since the response variable was well-being, it was important to know the relationship between this response variable and the other variables.

**Figure 10:**
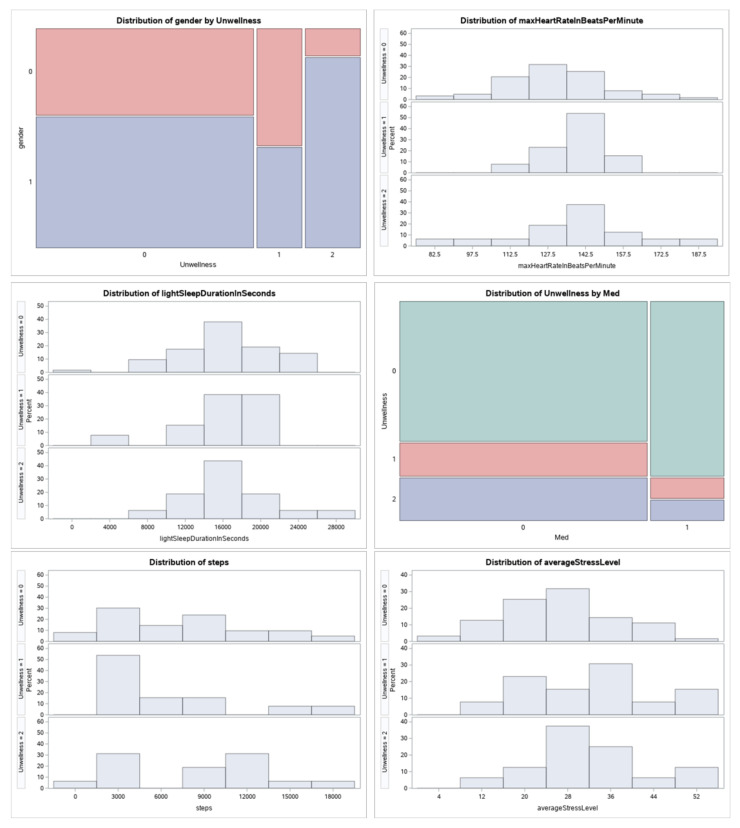
Relationship between variables.

Figure 10 shows the relationship between the response variable and some independent variables. It can show the proportion of a person’s well-being that can be bad (2). For example, people who do not exercise tend to have the worst well-being. Furthermore, according to the graph, women have a higher level of well-being than men.

After obtaining the information from the 22 users, various algorithms were used to extract the characteristics that impacted the model most to produce a more accurate classification.

### 4.5. Implementation of the Machine Learning Algorithm

From the entire dataset, approximately 68% of the cases have good well-being, making the dataset unbalanced; approximately 14% of the users have medium well-being and 17% of the users have bad well-being. This implies that there is not much information about not good well-being.

A feature selection algorithm was used to determine which combination was the most suitable to select which parameters have a higher impact on the model. As a result, when the best characteristics were chosen, ML techniques were used to see which features produced the greatest classifications. Due to the previous, the intelligent algorithms were run in different scenarios:

**Using univariate statistical tests**

The number of parameters used in each iteration was increased from 2 to 22, and the three different methods for feature selection using univariate statistical tests were run in each of the 20 iterations.

After these three feature selection methods, the ML techniques were run to obtain the best precision. As a result, the procedure illustrated in Figure 6 is repeated for each of the four feature extraction techniques, iterating between two variables and the entire number of parameters to determine which combination has the best accuracy.

Table 1 shows that performing feature selection increases accuracy. Furthermore, to simplify the model, the parameters height and weight were removed from the following extraction feature methods because those variables are already present in the IMC variable. Taking those two factors out increases the accuracy in the Random Forest. Furthermore, the height and weight variables were not used in the following extraction techniques.

In Table 2, an optimal accuracy of 88% was found, using only 16 out of the 20 variables that the final dataset had.

The accuracy was not improved for the *f_classif* method, as shown in the Table 3. The accuracy was 82%, which was not higher than the chi-squared test. The major improvement observed when applying the ANOVA-F test is that another ML method, Naive Bayes, also has relatively high accuracy.

In this work, different feature selection methods were implemented to improve the classification algorithm performance and to select the features that more impact the model (Table 4).

For the Chi-squared test, a pre-process of the inputs (X variables) was not needed. To obtain which variables have the highest variance, two lines of coding were necessary.

# Using the SelectkBest of the sci-kit Learn library

selector = SelectKBest(chi2, k = 16)

selector.fit(X, Y)

The selector variable specifies which variables were selected. Where X represents the 20 various parameters given in the previous Section 4.3, Y represents the response variable unwellness, and K represents the number of parameters the model must choose. It is crucial to note that this method was tested with several numbers of k (from 2 to 20), as shown in the accuracy tables, and K equal to 16 was chosen due to its high accuracy.

#### Random Forest

**Figure 11:**
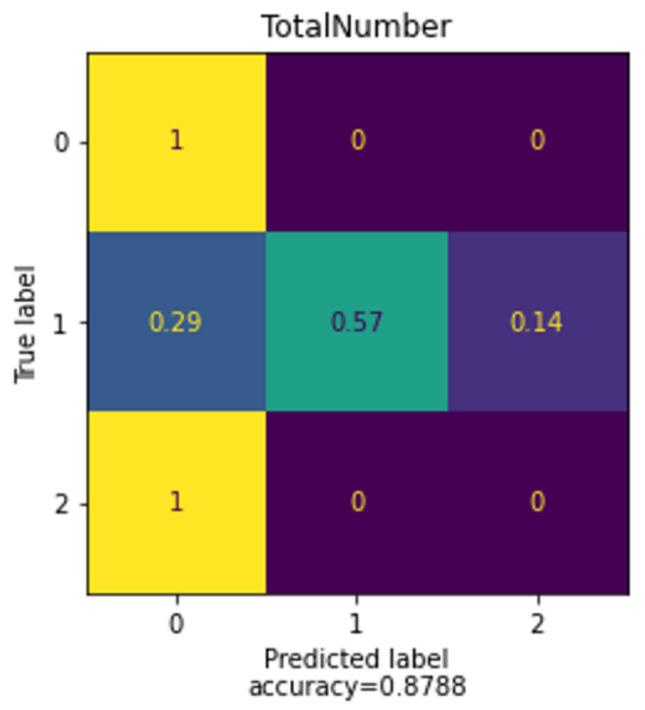
Confusion Matrix of Random Forest.

The confusion matrix of Figure 11 shows the specific accuracy of the Random forest method. When the user well-being is 2 (the worst well-being), the algorithm does not forecast well since the dataset is unbalanced, as indicated in the previous Figure 8. Only 16% of the algorithm dataset is labeled as having poor well-being; hence, the algorithm needs more data labeled as 2 to better classify.

**Figure 12:**
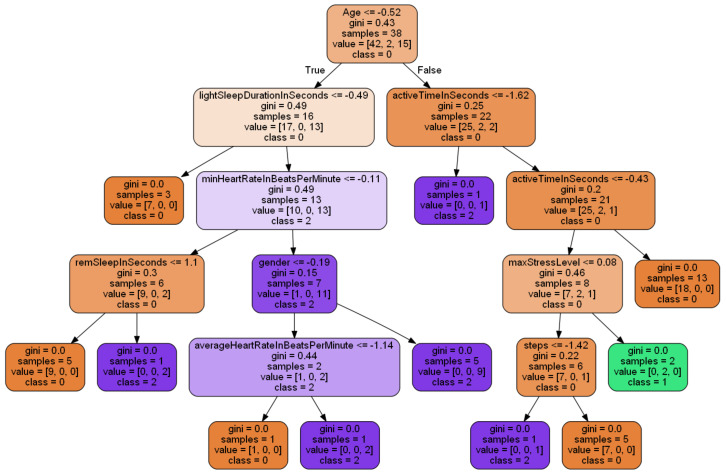
Best Tree.

Figure 12 illustrates the best tree generated using the Random Forest technique. The most crucial parameters that can influence a user’s health according to it are their age, time they spend light sleeping, and how much time they are active. It is possible to alter the overall well-being by adjusting these elements.

It was necessary to select, standardize, and train the dataset locally to optimize the cloud resources for the classification and feature selection. Consequently, the normalization model and the classification model obtained at the end of the training were saved in two different files to be uploaded to an S3 bucket. Subsequently, a Lambda function will fetch the files to restore and test the model with the new data (daily parameters for each user) and thus be able to predict the well-being of new input.

Two files are required to predict the well-being of the new input: the Random Forest training model and the standardization model. The standardization model was used to normalize the input based on the average and standard deviation of the inputs used to train the model (Random Forest training model). As indicated in the diagram, after standardizing the input X, the classification is made over the standardized $\hat{X}$.

**Figure 13:**
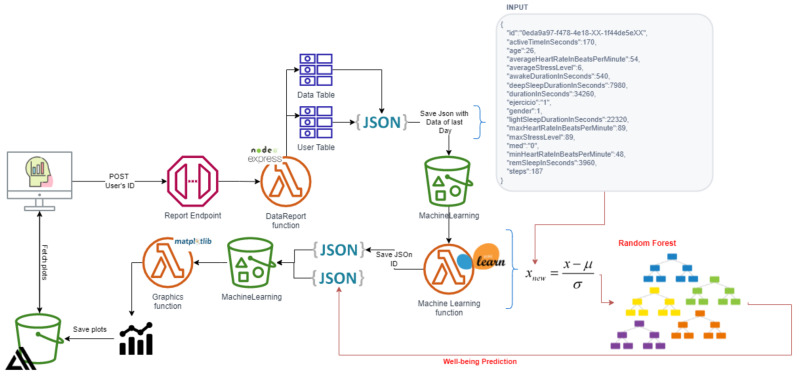
Classification diagram.

**Figure 14:**
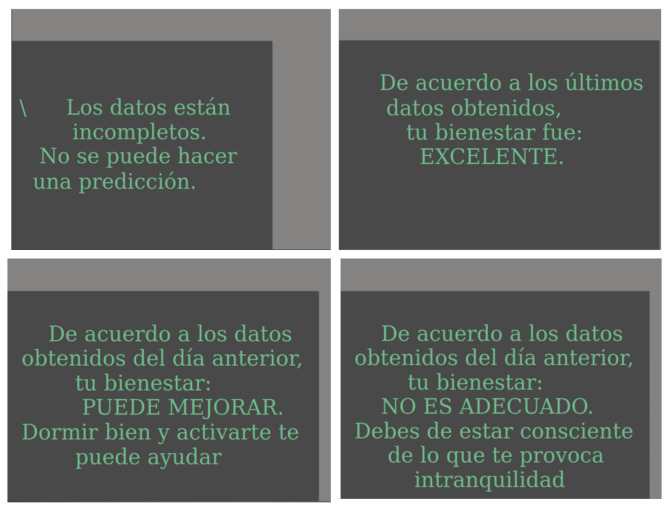
Classification images.

The classification also invoked the Graphic’s lambda function, which displays charts of several metrics measured by the smartwatch and the algorithm’s classification (Figure 13). The output classification is shown in the following four different figures:

The display Figure 14 can assist the user in better understanding how physical health might affect their well-being. As a result of LM Research’s forecasts, people can become more conscious of their habits and work to modify them to enhance their health.

## 5. Discussion

This project presents an intelligent system that monitors people’s physical and mental parameters to prevent a possible well-being crisis. In order to do this, monitoring will be performed through a combination of psychological and mental health data compilation that will be processed via machine learning algorithms for a prediagnosis of user well-being. For the mental health questionnaires, the user would log them through a webpage.

Even though several ML algorithms were tried, the algorithm performance could not be improved with an 88% of accuracy because the database was not balanced. In the end, the database is composed of 22 users and 35 features that evolve daily, with three different classes being unwellness or lack of well-being and has three values: 0 for excellent well-being, 1 for average well-being, and 2 for poor well-being. The majority of the system users present excellent well-being being difficult for the system to learn more about poor well-being.

In future work, the system should consider more cases of poor well-being, maybe by trying the system under circumstances where the user is not able to exercise and is under a lot of pressure. Furthermore, a factor that is not considered in this system is diet. Another interesting factor that was pointed out by this research is that women tend to have healthier habits in terms of exercise, which is reflected in their well-being index.

## 6. Conclusions

A health monitoring system comprised of smartwatches, communication APIs, and a web page was developed for this work; to acquire user physiological and mental parameters. Physiological parameters are essential for assessing a person’s state, especially if the person is experiencing an imbalance that disrupts the organism’s equilibrium. For those mentioned above, using all the data obtained and stored, a system was constructed to predict the person’s well-being daily using these factors, thereby preventing a probable crisis.

The study was conducted in three stages: The initial stage was data collection. At this step, the method and instrument for data acquisition are used to create a database. A key element that the project has benefited from is that smartwatches have grown in popularity as everyday items, making daily use very common among many people. Similarly, because the system was meant to be non-invasive and easily obtainable, a smartwatch was chosen as the instrument to assess each individual’s physiological data. Another significant advantage of implementing a smartwatch is that people use it for prolonged times since it is not uncomfortable for the user to wear it, resulting in more data recompilation. Garmin’s platform allows application developers to access all the data measured by the watches of the customers who gave data sharing consent; the project was uniquely based on data recompilation from these brands’ smartwatches. The second stage was establishing how the data would be handled; a REST API was developed to retrieve data from Garmin via a post request. This post request included a JSON file containing all the parameters measured by the watch daily. To accept the post request made by Garmin, Various Amazon Web Services technologies, such as Lambda functions and API gateway, were required for the API’s development. The third stage was how the physical data were integrated with the mental data in the same database. For the previous reason, the answers to the three different psychological questionnaires (after pre-processing them) were used to label all the physical data obtained by the watch as good, average, and low well-being. In order to gather these responses, it was essential to develop a dynamic web page that is entirely hosted in the cloud. Creating the website made it possible to establish a connection between the user’s data and LM Research. One of the webpage functionalities was that, after users provided consent to obtain Garmin data, a survey was sent to obtain questionnaire replies and profile data. Afterwards, the API was able to show all the data gathered into charts so that consumers could learn about their physical parameters over time. Finally, a labeled database was generated from all the acquired data. The database was used to train the algorithm to forecast each user’s well-being. This database comprised 96 entries and 23 variables, including wellness (the label data). The algorithms with the highest accuracy were the random forest and KNN algorithms. A random forest was selected to predict the well-being of each user every day.

As previously stated, the website can be considered the project’s foundation because it enables the collection of all the technologies and data required to achieve this project. Furthermore, it is crucial to highlight that everything was developed in the cloud, which enables the integration of various services to be more accessible, scalable to the project’s needs, and modular, which reduces resource usage.

A correlation between physical and mental data was confirmed through the analysis of the results. Moreover, this data can be measured by everyday items, such as wearables, as a tool to determine the overall well-being of a person. As of the last results, accuracy of 88% was obtained, confirming the existence of a correlation due to a predictive model, based on algorithm selection, that can correctly forecast data. It is a significant accomplishment because a well-being record can be created by measuring daily vital signs in collaboration with the smart system described in this thesis. The most significant parameters measured by wearables were determined using processing data methods, ML algorithms, and a random forest model, and people can now know which physical health factors should be prioritized to have good welfare.

With the results of this monitoring, people can better understand their health status by comparing obtained physiological data to measured physical data, resulting in long-term health improvement and a better understanding of their bodies. In other words, this can create awareness of how our overall health manifests in measurable data, with accessible instruments making a predictive analysis available.

By focusing on well-being, physical health outcomes can be improved. Given that increasing well-being can improve overall health, well-being interventions can be a practical way to create preventive medicine. Research into psychological well-being is starting to emerge. Nonetheless, assessing people’s well-being is critical because there is evidence that positive welfare and life evaluation can impact health and quality of life. Beyond diseases and physical health, healthcare systems should support methods for improving positive psychological states.

## Figures and Tables

**Table 1:** Table of accuracy with chi-squared.

Algorithm	2	3	4	5	6	7	8	9	10
Decision Tree	0.7	0.7	0.45	0.33	0.42	0.58	0.55	0.42	0.52
Random Forest	0.79	0.79	0.79	0.7	0.79	0.79	0.82	0.82	0.79
Naive Bayes	0.79	0.79	0.79	0.79	0.76	0.82	0.76	0.73	0.73
Lineal SVM	0.76	0.79	0.79	0.73	0.67	0.67	0.7	0.61	0.67
SVM	0.76	0.79	0.79	0.76	0.67	0.67	0.67	0.7	0.73
Neural network	0.76	0.21	0.39	0.36	0.61	0.73	0.67	0.61	0.64
KN Neighbor	0.76	0.79	0.42	0.61	0.67	0.61	0.64	0.58	0.64
NN with RELu	0.21	0.24	0.3	0.52	0.61	0.64	0.64	0.7	0.73
	11	12	13	14	15	16	17	18	19
Decision Tree	0.55	0.45	0.55	0.48	0.55	0.58	0.58	0.55	0.55
Random Forest	0.79	0.82	0.79	0.79	0.82	0.76	0.76	0.79	0.82
Naive Bayes	0.73	0.73	0.73	0.73	0.73	0.79	0.79	0.76	0.76
Lineal SVM	0.67	0.61	0.52	0.48	0.45	0.52	0.52	0.48	0.39
SVM	0.73	0.76	0.76	0.76	0.79	0.79	0.79	0.79	0.79
Neural network	0.61	0.52	0.61	0.55	0.58	0.61	0.48	0.55	0.61
KN Neighbor	0.55	0.61	0.52	0.61	0.58	0.61	0.64	0.67	0.48
NN with RELu	0.76	0.67	0.67	0.61	0.61	0.61	0.64	0.61	0.64

**Table 2:** Table of accuracy with chi-squared without height and weight.

Algorithm	2	3	4	5	6	7	8	9
Decision Tree	0.55	0.39	0.48	0.52	0.52	0.61	0.42	0.42
Random Forest	0.82	0.79	0.76	0.82	0.79	0.79	0.73	0.76
Naive Bayes	0.58	0.58	0.58	0.58	0.58	0.58	0.61	0.64
Lineal SVM	0.67	0.67	0.70	0.61	0.61	0.67	0.70	0.70
SVM	0.73	0.70	0.76	0.76	0.76	0.76	0.76	0.76
Neural network	0.55	0.27	0.58	0.48	0.52	0.52	0.48	0.45
KN Neighbor	0.58	0.61	0.58	0.58	0.58	0.61	0.45	0.67
NN with RELu	0.58	0.45	0.55	0.52	0.58	0.55	0.61	0.61
	10	11	12	13	14	15	16	17
Decision Tree	0.45	0.45	0.64	0.45	0.55	0.58	0.48	0.58
Random Forest	0.82	0.76	0.82	0.79	0.79	0.79	0.88	0.79
Naive Bayes	0.61	0.58	0.67	0.64	0.64	0.67	0.79	0.79
Lineal SVM	0.70	0.70	0.55	0.52	0.67	0.45	0.45	0.48
SVM	0.76	0.76	0.76	0.76	0.76	0.76	0.76	0.79
Neural network	0.52	0.52	0.58	0.58	0.58	0.58	0.58	0.52
KN Neighbor	0.61	0.61	0.58	0.64	0.55	0.55	0.64	0.55
NN with RELu	0.58	0.55	0.58	0.61	0.61	0.64	0.64	0.64

**Table 3:** Table of accuracy with f_classif without height and weight.

Algorithm	2	3	4	5	6	7	8	9
Decision Tree	0.79	0.48	0.64	0.70	0.58	0.45	0.55	0.48
Random Forest	0.79	0.76	0.73	0.82	0.82	0.82	0.79	0.82
Naive Bayes	0.79	0.79	0.82	0.82	0.82	0.79	0.79	0.82
Lineal SVM	0.76	0.79	0.70	0.67	0.73	0.79	0.73	0.73
SVM	0.79	0.76	0.70	0.70	0.70	0.73	0.70	0.70
Neural network	0.79	0.36	0.45	0.64	0.42	0.67	0.70	0.64
KN Neighbor	0.79	0.64	0.73	0.55	0.61	0.73	0.67	0.67
NN with RELu	0.33	0.52	0.58	0.55	0.64	0.67	0.73	0.73
	10	11	12	13	14	15	16	17
Decision Tree	0.48	0.58	0.55	0.58	0.52	0.58	0.52	0.45
Random Forest	0.79	0.76	0.79	0.76	0.82	0.79	0.82	0.79
Naive Bayes	0.76	0.79	0.79	0.73	0.79	0.79	0.79	0.79
Lineal SVM	0.67	0.55	0.52	0.48	0.42	0.42	0.45	0.48
SVM	0.76	0.76	0.76	0.79	0.79	0.79	0.79	0.79
Neural network	0.61	0.58	0.58	0.45	0.64	0.55	0.61	0.52
KN Neighbor	0.52	0.55	0.61	0.70	0.67	0.67	0.70	0.73
NN with RELu	0.70	0.67	0.61	0.61	0.61	0.64	0.61	0.64

**Table 4:** Table of accuracy with mutual_info_classif without height and weight.

Algorithm	2	3	4	5	6	7	8	9
Decision Tree	0.39	0.55	0.42	0.52	0.55	0.55	0.42	0.45
Random Forest	0.79	0.82	0.79	0.79	0.79	0.82	0.82	0.82
Naive Bayes	0.79	0.64	0.58	0.64	0.76	0.79	0.61	0.79
Lineal SVM	0.76	0.76	0.76	0.76	0.79	0.70	0.67	0.67
SVM	0.79	0.67	0.70	0.70	0.76	0.73	0.73	0.76
Neural network	0.36	0.24	0.52	0.58	0.55	0.61	0.39	0.52
KN Neighbor	0.79	0.55	0.58	0.61	0.58	0.58	0.52	0.52
NN with RELu	0.21	0.58	0.42	0.42	0.55	0.58	0.45	0.42
	10	11	12	13	14	15	16	17
Decision Tree	0.48	0.58	0.48	0.48	0.58	0.48	0.45	0.58
Random Forest	0.79	0.79	0.82	0.79	0.79	0.79	0.82	0.79
Naive Bayes	0.79	0.76	0.76	0.79	0.73	0.76	0.76	0.79
Lineal SVM	0.64	0.61	0.61	0.55	0.61	0.55	0.52	0.48
SVM	0.76	0.76	0.76	0.79	0.76	0.76	0.79	0.79
Neural network	0.45	0.48	0.45	0.70	0.48	0.64	0.55	0.52
KN Neighbor	0.64	0.58	0.42	0.55	0.36	0.48	0.52	0.61
NN with RELu	0.48	0.48	0.58	0.64	0.52	0.61	0.64	0.64

## Data Availability

Not applicable.

## Abbreviations

The following abbreviations are used in this manuscript:

Q-LES-Q	Quality of of Life Enjoyment and Satisfaction)
DASS-21	Depression Anxiety Stress Scale 21
ML	Machine Learning
GPRS	General Packet Radio Service
BDM	Blood-Glucose Monitor
ECG	Electrocardiogram
AWS	Amazon Web Services
MMASH	e Multilevel Monitoring of Activity and Sleep in Healthy
